# The Physiological Roles of the Exon Junction Complex in Development and Diseases

**DOI:** 10.3390/cells11071192

**Published:** 2022-04-01

**Authors:** Shravan Asthana, Hannah Martin, Julian Rupkey, Shray Patel, Joy Yoon, Abiageal Keegan, Yingwei Mao

**Affiliations:** Department of Biology, Pennsylvania State University, University Park, PA 16802, USA; sfa5385@psu.edu (S.A.); hpm5171@psu.edu (H.M.); jmr7124@psu.edu (J.R.); spatel@psu.edu (S.P.); jfy5125@psu.edu (J.Y.); ark5729@psu.edu (A.K.)

**Keywords:** RBM8A, MAGOH, EIF4A3, MLN51, neurodevelopment, NMD, mRNA

## Abstract

The exon junction complex (EJC) becomes an increasingly important regulator of early gene expression in the central nervous system (CNS) and other tissues. The EJC is comprised of three core proteins: RNA-binding motif 8A (RBM8A), Mago homolog (MAGOH), eukaryotic initiation factor 4A3 (EIF4A3), and a peripheral EJC factor, metastatic lymph node 51 (MLN51), together with various auxiliary factors. The EJC is assembled specifically at exon-exon junctions on mRNAs, hence the name of the complex. The EJC regulates multiple levels of gene expression, from splicing to translation and mRNA degradation. The functional roles of the EJC have been established as crucial to the normal progress of embryonic and neurological development, with wide ranging implications on molecular, cellular, and organism level function. Dysfunction of the EJC has been implicated in multiple developmental and neurological diseases. In this review, we discuss recent progress on the EJC’s physiological roles.

## 1. Introduction

Since the 1960s, the complex nature of the spliceosome in mRNA metabolism has been a focus of intense molecular and biochemical research [1,2]. At the turn of the century, exon-specific splicing associated factors were investigated as enhancers of endogenous alternative splicing, resulting in growing mechanistic insight into the differential processing of pre-mRNA transcripts [3,4,5,6,7]. In 2001, seminal publications implicated both RBM8A (also named Y14) and MAGOH as core components of the EJC [8,9]. Soon after, the core components EIF4A3 and MLN51 (also called CASC3) were defined and corroborated as significant components of the core EJC [10,11,12,13,14]. However, later studies demonstrate that MLN51 is a peripheral EJC factor with distinct function from the other 3 core factors (RBM8A, MAGOH and EIF4A3) [15,16,17]. Other auxiliary factors, such as CWC22, ALYREF, Acinus, RNPS1, SAP18, UPF3, PYM, join with EJCs and play critical roles in subsequent postsplicing functions [18,19,20,21,22,23,24,25,26,27,28,29,30,31,32]. Here, the EJC emerges as a crucial regulator of early gene expression, acting on a multifarious set of mRNA pathways to directly control mRNA splicing, export, translation, and degradation. Cumulatively, the EJC is composed of this trimeric core together with peripheral components whose structural integration is activity dependent [17,33,34,35,36]. EJC components have frequently been discussed using different names and these aliases are listed in Table 1.

### 1.1. Formation of the EJC

**Figure 1:**
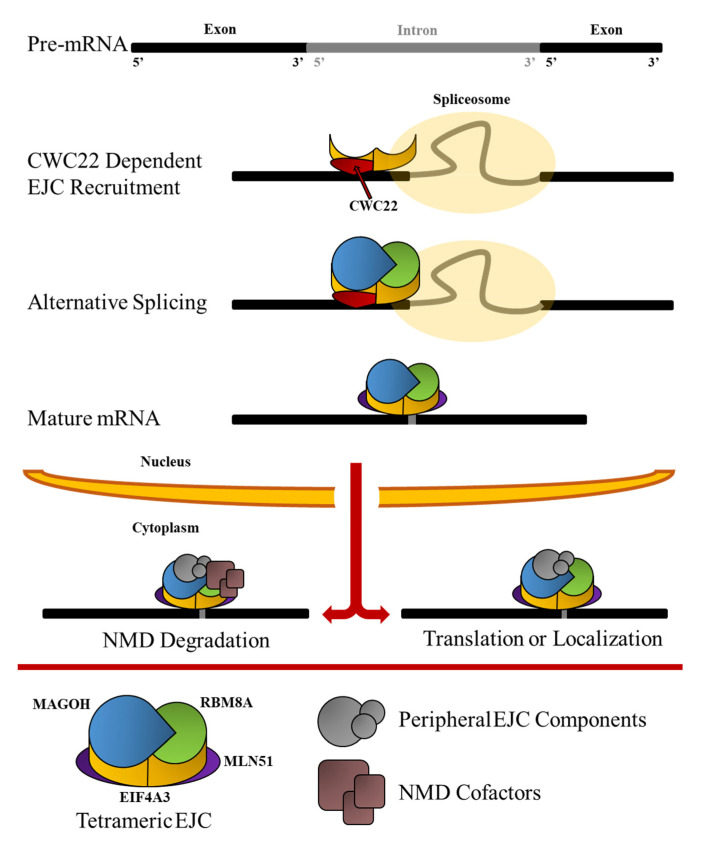
Graphical representation of the formation and function of the EJC. Splicing factor CWC22 bridges spliceosome activity to EJC formation by recruitment of EJC core component EIF4A3. Spliceosome formation is completed concurrently with immature EJC formation as the MAGOH-RBM8A heterodimer binds to EIF4A3. After splicing, MLN51 completes the mature tetrameric EJC core, and the EJC-associated mRNA is exported from the nucleus to the cytoplasm. Peripheral EJC components and NMD cofactors mediate the differentially factor-specific progression to nonsense mediated decay or downstream pathways further in the cytoplasm including translation and mRNA localization.

It is known that the EJC displays a high affinity for mRNA transcripts requiring translation to render EJC and mRNA mutual dissociation [44]. This stable association is described as a lock-on function delivered by EIF4A3 DEAD-box domain and ATPase activity, further stabilized by a heterodimer of RBM8A and MAGOH (Figure 1) [11,45]. In the early stages of the splicing process, a trimeric immature pre-EJC is therefore composed, and upon exon ligation MLN51 completes the complex forming the mature and tetrameric EJC. Furthermore, MLN51 stimulates both ATPase and RNA helicase activity, although only the former is inhibited by the RBM8A/MAGOH heterodimer, functionally locking EIF4A3 onto the mRNA transcript by preventing the release of hydrolyzed ATP [11,46]. Assembly of the EJC takes place on the mRNA about 20–24 nucleotides upstream of the exon-exon junction, and occurs in a regimented splicing-dependent manner, not requiring a specific sequence to attach [47,48]. Recruitment of each of the EJC components into a mature complex is feasible in in vitro conditions amenable to RNA and ATP, however, physiological assembly is highly splicing-dependent [49]. In fact, a direct link between EJC deposition and splicing activity is provided by the spliceosome protein CWC22, an essential splicing factor and binding partner of EIF4A3. CWC22 protein contains a MIF4G domain that upon binding maintains EIF4A3 in an open binding conformation [45,50]. In addition to its abundance in the spliceosome, CWC22 knockout significantly decreases spliceosome efficiency demonstrating its necessity in splicing.

### 1.2. The EJC at the Cellular Level

Within the nucleus, the EJC therefore influences splicing events. Studies of core component orthologues in *C. elegans* and *Drosophila* demonstrate that dysregulation due to variable depletion or knockdown of either RBM8A, EIF4A3, or MAGOH results in nuclear RNA leakage and alters transcriptome wide alternative splicing products [51,52]. Studies further identify additional deregulation of neural microexon networks in mammalian cells [53,54,55]. Hence, it has been hypothesized that the EJC plays a critical role in the temporal order of splicing events [54,55]. Early structural insight into the inner and outer shell composition of the EJC demonstrated that the different mutations of outer shell, activity-dependent peripheral factors could be responsible for the location, mobility, and enrichment of the EJC in mRNA perispeckles [56,57]. Once in the cytoplasm, the EJC accompanies the mRNA transcripts to translation sites, at which point EJC disassembly starts. Spliced mRNAs are translated at a higher volume due in part to the EJC’s almost universal presence on spliced mRNAs [58,59,60]. The interacting partner of RBM8A and MAGOH, PYM, plays a significant role in structurally bridging stable interactions between the core EJC and ribosomal complexes in addition to regulating normal homeostasis of the EJC [32,61]. Cellular localization of mRNA is regulated comprehensively, and the EJC has consistently been implicated in this process as well. MLN51 interacts with EIF4A3, a component of the *Oskar* messenger RNP complex essential for *Drosophila* mRNA localization and degradation targeting in mammals [14,62]. Overexpression of MLN51 in *Drosophila* has been associated with P-body disassembly and novel RNA granule formation distinct from stress granules [63]. 

The EJC factors play a critical role in nonsense mediated mRNA decay (NMD), a well conserved RNA surveillance mechanism specialized in identifying and degrading aberrant mRNAs containing premature termination codons (PTCs) [64]. NMD mechanism also targets several other types of normal mRNAs, such as mRNAs with long 3′ untranslated region (3′UTR > 1 kb) and with 5′ upstream open-reading frames (uORF) [65,66]. The NMD pathway controls the quality of post-transcriptional mRNA and regulates alternative splicing products, and there has been tremendous interest due to well established links between NMD, genetic disease, and cancer [67,68,69,70]. Individual EJC core components were gradually found to be associated and interactive with key NMD proteins, such as the Up Frameshift (UPF) proteins, UPF1, UPF2, UPF3A, and UPF3B [12]. Early mechanistic insight demonstrated that both EJC-dependent and -independent NMD pathways, occur across differential cofactor requirements.

### 1.3. Overview of the EJC’s Position in Development

Given the EJC’s considerable influence on mRNA abundance, there are significant implications on the developing organism. NMD core component UPF proteins in the EJC dependent pathways were first linked to embryonic development, early patterning, and viability of animal embryos [71]. Conclusive evidence directly linking the EJC to early development came when MAGOH and RBM8A haploinsufficiency was shown to result in mouse microcephaly with follow up studies indicating high EJC enrichment in neural progenitors and the EJC being critical for cortical development [72,73,74]. The cellular environment is similarly shown to be influenced by EJC dysfunction, and several key publications indicate implications on neuronal activity [75] and mouse behavior [76].

RBM8A overexpression in the adult mouse hippocampus generates distinct anxiety and autism spectrum disorder (ASD)-like behaviors [76] while EIF4A3 knockdown in hippocampal neurons increases synaptic strength via AMPA receptor abundance at excitatory synapses [75]. The EJC is a regulator of cell cycle and apoptosis progress where RBM8A and MAGOH diametrically interact with the STAT-3 cancer target pathway [77,78,79]. In human disease, EJC dosage is clearly relevant as evidenced by study of the 1q21.1 chromosome region including the *RBM8A* gene. Thrombocytopenia with Absent Radius (TAR) syndrome is characterized as a blood and limb disorder in addition to neurodevelopmental phenotypes at an increased incidence. TAR syndrome results from compound mutations of a 1q21.1 null deletion and rare SNPs in the regulatory region of *RBM8A* [80,81]. Richieri–Costa–Pereira (RCP) syndrome patients present with learning and language deficits in addition to abnormal craniofacial and limb features. RCP syndrome arises in part due to repeat expansion in the *EIF4A3* gene [82]. Mutations in crucial NMD components, such as *UPF3B*, have been identified in a diversity of neurological diseases ranging from ASD to schizophrenia [83].

Thus, the EJC emerged as a crucial molecular target to investigate the physiological function of NMD, as well as many other roles in the mammalian cell. We aim to assess the physiological roles of the EJC in development and disease by investigating the broad scale of EJC function ranging from molecular and cellular regulations to organismal development and diseases.

## 2. The Functions of EJC in mRNA Translation, Localization, and NMD

### 2.1. The mRNA Localization and Translation

Messenger RNAs (mRNAs) are transported to cytoplasm and bind to ribosomes where proteins are produced [84,85]. The transportation and functions of mRNA are regulated by the EJC and NMD through localization, transcription, translation, and splicing [84,85]. Distribution of mRNAs in the cytoplasm is not random, but close to specific subcellular areas and structures that are associated with their protein metabolism and functions [84,85]. Bacteria, yeast, and mammalian cells all have specific mRNA localizations [85]. Untranslated mRNAs can be stored in P-bodies or stress granules in response to aversive environments [86]. Some mRNAs can also be locally translated, delivering mature polypeptides to specific cellular locations, and contribute to a multitude of cellular processes [84], including asymmetrical cell division, cell polarity and motility, cell migration, cell fate, and neuronal synaptic plasticity [87].

The mRNA localization is highly correlated with the encoded proteins where their functions is performed [88]. Location specificity for mRNAs can be achieved through multiple processes. Most mRNAs are actively transported by cytoskeleton motor proteins, while some are passively diffused and are either locally compartmentalized and/or are locally shielded from degradation by regulatory factors [89,90,91]. These regulatory molecules can be cis- or trans-acting [84]. Cis-acting elements found within the 3′UTR define the location and type of information they can carry to localize the given transcripts. They can bind trans-acting RNA binding proteins (RBPs) that mediate RNA metabolism and act as regulators of post-transcriptional gene expression [92,93,94]. Nascent polypeptides can also be actively moved to target location along with mRNAs [84]. Recent studies have investigated this process through the signal recognition particle (SRP) that binds nascent signal peptides. This action was suggested to inhibit translational elongation and mediate anchoring of the nascent polysomes to the SRP receptor on the ER, where translation elongation resumes but the role of translation in localization of some mRNA types was unknown [95,96,97].

EJC factors regulate mRNA distribution. In *Drosophila*, Y14 (RBM8A) and Mago control *oskar* RNA shuttling to the posterior of the oocytes, which is crucial for germline and abdomen formation during embryo development [98,99]. Upregulation of *Drosophila* PYM dissociates EJC from *oskar* RNA and leads to mislocalization of *oskar* [61]. Interestingly, EJC proteins accumulate at the basal body of cilia in mouse neural stem cells and human retinal pigment epithelial (RPE1) cells [100]. Trafficking of *NIN* mRNA, encoding a core component of centrosomes, towards centrosome is dependent on EJCs [100]. Mitotic centrosome mRNA localization was first observed as a way of asymmetrical mRNA segregation for developmental patterning in mollusk embryos [101]. Further studies of *Drosophila* and zebrafish embryonic development have also found mRNAs associated with centrosome protein [102,103]. Another study investigated mRNA localization and found three mRNAs localized to mitotic centrosomes with their proteins (HMMR, NUMA1, and ASPM), indicating that these proteins are locally translated during mitosis at the spindle poles [85]. This research also observed polysome aggregates that act as specialization complexes of nascent protein translation. Fifteen of the discovered foci were identified as p-bodies, while four distinct mRNAs (ASPM, BUB1, DYNC1H1, and CTNNB1/β-catenin) displayed distinct, specialized translational abilities [85]. When bound to β-catenin, APC, AXIN, GSK3, and CK1α form a destruction complex of β-catenin to phosphorylate β-catenin and proceed it to degradation by the proteasome [85]. Interestingly, β-catenin mRNA foci also contained the factors of the destruction complex, indicating the degradative function of this complex [85]. This translational complex is indicative of a gene regulatory mechanism involved in protein degradation [85]. The function of the other three complexes is unknown, but it was suggested that they could be involved in co-translational protein complex assembly or localization of chaperones or modification enzymes [85]. Further research into mRNA localization and these complexes related to EJCs could lead to important discoveries for utilization of such mechanisms in diseases and disorders affected by these components.

### 2.2. Implications of MLN51 in P-Body Formation

In 2014, MLN51, a peripheral component of the EJC mainly found in the cytoplasm, was identified as a component of p-bodies through interactions with polysomes [63]. Overexpressed MLN51 localizes in stress granules and in small MLN51-induced granules (SMIGs). MLN51 overexpression was shown to induce SMIGs and subsequently disassemble mammalian p-bodies in a microtubule-dependent manner [63]. It was suggested that removing repressed mRNAs from p-bodies via transport factor action of MLN51 could cause p-body disassembly. RBM8A overexpression has previously been identified in upregulation of p-bodies following pioneer rounds of translation. MLN51 interacted with p-body utilizing a separate region of MLN51 different in EJC complex recruitment [63]. This unique separate region of MLN51 and the ability to produce p-body from a MLN51 variant mutated in EJC-binding domain, support an important role of MLN51 interacting with mRNAs independent of the EJC [63]. MLN51 is also found to be overexpressed in malignant breast cancer cells. MLN51 is found in the same chromosomal region as the *c-Erbb2* oncogene that is implicated in cancer pathogenesis [63]. P-bodies are disassembled in HER2+ breast cancer cells overexpressing MLN51, suggesting that p-body status could correlate with cancer malignancy [63].

### 2.3. The EJC Serves as a Crucial Link between Splicing and NMD

NMD acts to prevent the generation of deleterious truncated proteins. Premature termination codons (PTCs) in transcripts, derived from nonsense mutations and errors in splicing, can result in toxic proteins in the absence of NMD [104]. EJC-dependent NMD has been shown to be highly efficient [104]. EJC-independent NMD occurs as a response to other messenger ribonucleoprotein (mRNP) features [104]. Recent investigations have revealed this pathway’s important role in global transcriptome regulation [104]. Splicing is an integral part of both the EJC and NMD, as pre-mRNA splicing is required for EJC-dependent NMD where the EJC binds to the mRNA once splicing has occurred [104]. Splicing can influence NMD efficiency, but the mechanisms for how this is done require further investigation [104]. Recent studies of NMD efficiency have shown that overexpression of the splicing factor SRSF1 increases NMD efficiency by acting as a positive regulator of NMD through enhancing UPF1 binding to mRNA in the nucleus, providing a possible answer to the underlying mechanism of splicing and NMD [104]. RNPS1 protein, a pre-mRNA splicing activator in vitro, is a peripheral factor of the EJC [105]. Recent findings have shown that knockdown of RNPS1 causes abnormal splicing patterns, indicating that *Rnsp1* is an important quality control factor for mRNAs and cell division [105].

## 3. The Functions of EJC in Development

### 3.1. The EJC Regulates Neural Proliferation and Differentiation

The exon junction complex and its components have diverse roles in cellular differentiation and development. The EJC has a crucial role in the maintenance of stem and progenitor cells in planarians [106]. While it was not necessary for the differentiated cell response to amputation, EJC is necessary for maintaining the population of undifferentiated cells [106]. In mice, it has been demonstrated that haploinsufficiency in *Magoh*, *Rbm8a*, or *Eif4a3* is sufficient to cause microcephaly in mice, indicating that these components are necessary in neurogenesis [107]. MAGOH has been shown in neural progenitors, via live imaging, to delay mitotic progression, but not in post-mitotic neurons [108]. The effect of this in *Magoh*-deficient radial glial cells resulted in an increase of differentiated or apoptotic progeny [108,109]. Specifically, a delay of prometaphase creates an imbalance with an increase in the number of neurons and a decrease in the number of progenitor cells [109]. The role of *Magoh* in proliferation even extends to neural crest-derived melanocytes, where it regulates melanoblast proliferation [110]. A neuroblastoma cell study indicated that interactions between *Eif4a3* and *Cdc174* are associated with proper neuronal differentiation [111]. *Cdc174* mutations lead to nuclear aggregates that lead to apoptosis, manifesting as psychomotor development delay and hypotonia [111].

During embryonic brain development, *Rbm8a* is critical for proliferation, differentiation, and migration of both excitatory and interneuron progenitor cells [73,74]. The overexpression of RBM8A during development suppresses differentiation and increases proliferation in neural progenitor cells [74]. Proliferation is promoted by inhibiting exit from the cell cycle [74]. Haploinsufficiency of *Rbm8a* has been found to cause apoptosis and the manifestation of microcephaly in mice [73,112]. *Rbm8a* deficient cells have also been found to accumulate DNA damage which both decreased cell viability and the proliferation of neural stem cells. RBM8A was found to recruit Ku70/80 to sites of DNA damage during the cell DNA damage response [113]. *Rbm8a* mRNA is regulated by other factors such as miR-29a in retinal progenitor cells [113]. miR-29 is capable of repressing *Rbm8a* via 3′ UTR binding, which increases differentiation and decreases proliferation [114]. Additionally, the *Drosophila* orthologue of MLN51, *Barentsz*, has been found to promote neuromuscular synapse growth via increased activin signaling, in an EJC-independent manner [115]. Additionally, a correlation has been observed between autophagy in Alzheimer’s disease and *RBM8A*, indicating possible contribution to pathophysiology [116]. The processes of gene activation and alternative splicing are tightly regulated by splicing regulators in eukaryotic cells. This study supports a crucial role for EJC components in splicing a specific subset of introns.

### 3.2. Regulation of Splicing during Development

EJC factors sit on exon-exon junctions during splicing to control mRNA quality. *Drosophila* RBM8A and MAGO control *oskar* RNA localization thereby regulating oogenesis [98,99]. Similarly, RBM8A and MAGO are essential for *C. elegans* embryonic development and germline sexual switching. Knockdown of these two EJC factors by RNA interference leads to lethality [117]. In addition, *Drosophila* MAPK is required for epidermal growth factor signaling-mediated eye development. Suppression of MAGO, RBM8A and eIF4A3 causes defects in eye development [118]. Interestingly, Mago depletion does not affect the transcription or stability of MAPK mRNA but changes its splicing pattern thereby reducing the MAPK expression level [118]. Consistent with this notion, other essential developmental signaling pathways, such as Wingless/Wnt pathway, are regulated by EJCs. Dishevelled (Dsh) controls Wnt activation and interacts with a cell polarity protein disc large 1 (Dlg1). This Dsh-Dlg1 interaction prevents Dsh from lysosomal degradation. Interestingly, EJC modulates the splicing of Dlg1 thereby modulating Wingless/Wnt signaling pathway [119]. During mouse brain development, EJC levels are critical to maintain normal mRNA splicing. Either downregulation or upregulation of EJC factors can result in abnormal splicing events during embryonic brain development [74,107]. However, it remains to be determined what abnormal splicing events are caused directly by EJCs.

### 3.3. Control of mRNA Fate by the EJC during Neural Development

The localization of mRNA transcripts is important to normal neural events. In radial glial progenitors, it was found that specific mRNAs were locally translated at end feet [120]. mRNAs reached the end feet via the basal process. Proteins, such as FMRP, serve to transport certain transcripts, allowing for accumulation of specific transcripts that are crucial for proper neurogenesis [120]. Similarly, *Arc* mRNA accumulates in dendrites soon after it is transcribed where it is locally translated at synapses [121]. However, *Arc* mRNA is negatively regulated upon activation of NMDA receptor results in mRNA degradation [121]. The EJC has been implicated in mRNA localization from a study of mouse neural stem cells [100]. Depletion of EJC impairs proper centrosome organization and ciliogenesis, suggesting that EJC-dependent mRNA localization is necessary for normal neural stem cell division and brain development in mice [100].

The EJC component, EIF4A3 is also known to interact with *Arc* mRNA [122]. In adult Sprague Dawley rats, spatial exploration resulted in increased expression of *eIF4A3* mRNA in the dorsal striatum and hippocampus [122]. EIF4A3 interacts with *Arc* mRNA to regulate its level via EJC-dependent NMD [75,122]. This signifies EIF4A3 as a regulator of synaptic strength and GLUR1 AMPA receptor concentration in neurons [75]. The role of NMD in this process is confirmed in adult mice via disruption of UPF2 in neurons, which increases GLUR1 local synthesis in dendrites, along with decreased memory, learning, and synaptic plasticity [123]. Therefore, NMD downregulates GLUR1 via the downregulation of *Arc* mRNA [123].

### 3.4. Crucial Roles of NMD in Processing mRNAs during Development 

NMD control is essential for checking the quality of mRNA transcripts, in which high quality RNAs have a long half-life [124]. In *C. elegans,* the activity of NMD decreases as an organism ages [124]. Multiple pathways can regulate NMD. Changes in the composition of the EJC are associated with differential regulation of NMD activity, which leads to different branches of NMD pathways targeting specific subsets of mRNAs. Interestingly, the peripheral EJC factor, RNSP1, form a complex with EJC core that is distinct from the MLN51-EJC core complex. RNSP1-EJC core interaction allows NMD to target most transcripts in a cell. When RNSP1 is switched to MLN51, the activity of NMD decreases and only a selective set of transcripts are targeted by NMD [24]. Consistently, different branches of NMD have been observed in NMD factors, UPF2 and UPF3, in which depletion of UPF2 and UPF3 has no effects, supporting that UPF2- and UPF3-independent NMD branches exist [125,126,127]. Consistently, *UPF3B* gene carrying PTC is susceptible to NMD-mediated degradation that leads to no *UPF3B* protein production, confirming that UPF3-independent NMD can eliminate mutated *UPF3B* transcript [128].

NMD is also linked to synapse morphology and function via its regulatory role of mRNA translation. This is supported by mutations in NMD components, SMG1, SMG6, and UPF2, resulting in altered synapse structure and reduced strength of neurotransmission [129]. Knockout of essential NMD endonuclease, Smg6, in neural progenitor cells (NPCs), causes perinatal lethality as results of decreased cortical NPCs and abnormal interneuron development [130]. Clearly, targeting individual NMD factors or manipulation of NMD activity in embryonic stem cells affects cell fate determination and differentiation processes [131,132,133,134]. Consistently, Upf2 is required for normal liver development/regeneration [135] and Sertoli cell development and male fertility [134], supporting an essential role of NMD factors in normal organismal development.

### 3.5. Crucial Roles of NMD in Diseases

The accumulating evidence shows defective NMD in cellular functions contributes to neurodevelopment disorder. Deletion of the *UPF3B* gene is associated with childhood onset schizophrenia [136,137].Throughout development UPF3B expression and localization vary [136]. During brain development, *Upf3b* regulates multiple cellular processes [136,138]. Proper differentiation of neural progenitors is dependent upon *Upf3b* while in post-mitotic neurons, *Upf3b* regulates neurite growth [136]. Consistent with this notion, in UPF3B-null mice, neural stem cell proliferation is increased but differentiation is decreased. These mice showed specific memory, sensorimotor gating, and dendritic maturation defects [139]. Single-cell RNA-sequencing (scRNA-seq) analysis further revealed that UPF3B knockout significantly affects olfactory receptor gene expression in mature olfactory sensory neurons [140].

UPF2 variants associated with speech and language deficits manifest through UPF2-dependent NMD inhibition [141]. Conditional knockout of *Upf2* in mouse brain leads to deficits in memory, social behavior, and long-term potentiation [123,141]. Furthermore, an immune response resulted and its treatment by anti-inflammatories alleviated the deficits [141]. Amyotrophic Lateral Sclerosis (ALS) familial mutations in the FUS protein impede the self-regulation of NMD, which leads to excessive targeting of cellular transcripts [142]. Furthermore, viral infections inhibit NMD in order to protect viral transcripts from degradation [143]. Upon infection, viral capsid protein of Zika virus binds to UPF1 and targets it for degradation, allowing for pathogenesis to occur in neural progenitor cells and leading to phenotypes like microcephaly [143].

MiR-128 binds to UPF1 and MLN51 mRNAs and inhibits NMD. Therefore, This is relevant for neuronal cell differentiation and brain development as miR-128 is utilized to increase levels of transcripts related to development and neural function [144]. NMD can also be regulated in neurons by an RBP, NOVA [145]. These proteins introduce PTC via alternative splicing to regulate cytoplasmic mRNA levels. Interestingly, these regulators act on protein levels after seizures, particularly in proteins that inhibit seizures and maintain homeostasis [145]. Spontaneous epilepsy was observed in mice with NOVA haploinsufficiency. NMD is often altered by mutations in component proteins [141,142]. Upon deletion of EIF4G1 and EIF4G3 using CRISPR-Cas9, mice showed deficits in learning, memory, and social behavior, along with altered synaptic plasticity in the hippocampus [146].

In patients with copy number variant gains of NMD components, NMD over-activation likely harms development and neuronal function and gives rise to intellectual disabilities [147]. Mutations in normal gene products can introduce PTCs to transcripts leading to NMD based degradation. For example, PTCs in the *NCL* gene cause Batten disease, an autosomal recessive neurodegenerative disease [148]. Likewise, a PTC mutation in GABRA1 results in a lack of a functional receptor and epilepsy [149]. NMD targets the majority of PTC-containing GABRA1 mRNA, those that are translated into the ER are subsequently eliminated by endoplasmic reticulum associated degradation [149]. Disruption of translation itself is associated with abnormal function and leads to ASD-like phenotypes [146]. However, PTC read-through drugs, such as anti-sense oligonucleotides, are being explored to treat PTC-based pathologies [148,150].

## 4. EJC Components Are Implicated in Phenotypically Diverse Genetic Disorders

The EJC has been implicated in a variety of disorders of genetic origin, although below we will focus on the most well characterized human implications (Table 2).

### 4.1. The Importance of RBM8A in the Developing Organism

1q21.1 deletion is associated with neurodevelopmental disorders, congenital heart disease, dysmorphic features, and thrombocytopenia absent radius (TAR) syndrome [80,158,159,160]. Phenotypes of individuals with 1q21.1 copy-number variants show motor and cognitive deficits [158]. Whereas duplication carries had a higher prevalence of motor impairments and ASD; deletion carriers had increased rates of microcephaly [158]. Epileptic-dyskinetic encephalopathy is caused by *EEF1A2* mutants that impede a cell’s ability to cope with cytotoxic stressors [161]. In early brain development, it is possible that synaptic protein synthesis is impaired [161]. Microdeletion of proximal 1q21.1 is mainly associated with TAR syndrome due to a deletion of the *RBM8A* gene and often referred to as the TAR region. TAR syndrome is congenital and is diagnosed for low platelet count in early age or reduction of megakaryocytes in the bone marrow. Patients with TAR syndrome often show a wide range of skeletal abnormalities from preserved thumbs, radial anomalies, renal anomalies, lower-limbs anomalies to cow’s milk intolerance [162,163,164].

TAR syndrome follows autosomal recessive inheritance and de novo 1q21.1 microdeletion is frequently found in the affected individuals, it suggests that 1q21.1 microdeletion is not sufficient to cause the syndrome, it requires an additional causative allele [81]. A family genome study monitored the exome sequences of the affected and healthy individuals. The study found that the patients often carry a low-frequency single-nucleotide polymorphism (SNPs) in the 5′-untranslated region (UTR) of the *RBM8A* gene or an unknown SNP in the first intron of the gene along with the 1q21.1 deletion [81]. The *RBM8A* gene appears a strong contributor to the TAR syndrome in that the 5′UTR SNP was also found in the affected without the 1q21.1 deletion [80,81]. Moreover, the two noncoding SNPs resulted in reduction of *RBM8A* transcription in vitro and reduced protein-RBM8A expression, suggesting TAR results from insufficiency of the protein-RBM8A [80]. This dosage-effect is significant as RBM8A plays a significant role in cell cycle regulation, however, the mechanisms by which candidate SNPs reduce the levels of protein-RBM8A in platelets are not well understood [165].

Furthermore, *RBM8A* plays an evolutionarily conserved role in the development of reproductive organs and gametes alongside *Magoh*. In *Drosophila* oocytes, RBM8A and MAGOH form a complex with *Ranshi* to localize *Oskar* mRNA to the posterior pole during oocyte differentiation [166]. In *C. elegans* germ cells, RNAi inhibition of *Rbm8a* and *Magoh* resulted in embryonic lethality and masculinization of the hermaphrodite germline [117]. Similarly, in the trematode *Schistosoma Japonicum*, RNAi inhibition of *Rbm8a* led to dysplasia of the testes, eggs, and vitellaria [167]. Because mutations in other splicing factors have been shown to cause masculinization, *Rbm8a* is likely involved in sexual differentiation by regulating alternative splicing events [167]. Clinically, variations of *RBM8A* have been associated with fusion disorders of the Müllerian ducts and Mayer–Rokitasnky-Küster–Hauser syndrome. Mayer–Rokitasnky–Küster–Hauser syndrome and some TAR patients exhibit congenital absence of the uterus and upper part of the vagina, indicating *RBM8A* plays a mechanistically unclear role in the development of female sexual organs [151].

### 4.2. EIF4A3, MAGOH, and Peripheral Components’ Differential Associations in Human Disease

The EJC factor *EIF4A3* has also been implicated in diseases, including Richieri–Costa Pereira syndrome, a rare acrofacial dystosis. A noncoding expansion in *EIF4A3* identified in Richieri-Costa-Pereira patients has been predicted to interfere with UPF3B to cause craniofacial and limb underdevelopment [82]. Later investigations into the mechanistic implications of this expansion revealed that while Richieri-Costa-Pereira syndrome is an autosomal-recessive disease, *EIF4A3* haploinsufficient mouse embryos have altered micrognathia and altered mandibular fusion [168]. In Richieri–Costa Pereira patients, neural crest cell (NCC)-derived mesenchymal stem-like cells demonstrated premature differentiation, and underdeveloped cartilage was observed in vivo mouse models, indicating *EIF4A3* interacts with *Upf3b* to regulate NCC-derived mesenchymal cell migration and differentiation [168].

Haploinsufficiency of either *Eif4a3*, *Rbm8a*, or *Magoh* has been linked to microcephaly through converging regulation of the p53 pathway, and p53 ablation has been shown to rescue the microcephaly phenotype in mice [107]. In addition to the core EJC factors, UPF1 has been mechanistically implicated in a genetic muscular disorder. Misexpression of the DUX4 gene, which has been associated with facioscapulohumeral muscular dystrophy, results in proteolytic degradation of UPF1 in myoblast cultures and eventual NMD inhibition and cellular apoptosis [169]. Because NMD destabilizes misexpressed DUX4 mRNA, UPF1 degradation causes a positive feedback loop that results in the accumulation of DUX4 [169].

## 5. Conclusions

In summary, it is evident that the cellular processes that the EJC regulates play a crucial role in organismal development. Precise control over splicing, mRNA localization, and degradation by EJC tightly regulates embryonic and neurodevelopment, with further implications on human diseases. In vitro system, model animals, and human genetic studies have together accumulated powerful insights into the physiological roles of EJC, yet many questions remain open. The whole life cycle of the EJCs and their downstream pathways remain to be elucidated. Moreover, full investigation of EJC core and peripheral components in a great diversity of mammalian models offers the opportunity for a deep mechanistic understanding of the pathogenesis of related human disorders.

## Figures and Tables

**Table 1:** Common aliases used to describe each of the major EJC components discussed in this paper as RBM8A, MAGOH, EIF4A3, and MLN51. Included are the model organisms used to find in which species the alias was first identified or used with corresponding references.

EJC Component	Common Aliases	Model Organism	Reference
RBM8A	Y14	*Homo sapiens*	[37]
	Tsunagi	*Drosophila melanogaster*	[38]
	BOV 1	*Saccharomyces cerevisiae*	[39]
MAGOH	Mago-Nashi	*Drosophila melanogaster*	[40]
EIF4A3	DEAD Box Protein	*Mus muculus*	[13]
	NMP 265	*Homo sapiens*	[41]
	DDX48	*Homo sapiens*	[42]
MLN51	CASC3	*Homo sapiens*	[43]
	BTZ	*Homo sapiens*	

**Table 2:** Core EJC Components and associated human disorders. Methodology in which the association was made between the EJC core component, and the human disorder is summarized accordingly as the tool employed in discovery. References are provided for the publications establishing the relevant association.

EJC Component	Implicated Human Disorders	Tools Employed in Discovery	References
RBM8A	Thrombocytopenia-Absent Radius Syndrome (TAR)	Genetics analysis	[80,81]
	Autism Spectrum Disorder	Mouse RNAi/Over-expression	[76]
	Mayer–Rokitansky–Küster–Hauser syndrome	Genetic variation analyses	[151]
	West Nile Virus	RNAi	[143]
	Glioblastoma	RNAseq Analysis	[152]
	Hepatocellular Carcinoma	Gene Expression Profiling Interactive Analysis	[153,154]
	Cervical Cancer	Microarray Gene Analysis	[155]
	Alzheimer’s Disease	GSEA and Identification of D.E.G.’s	[116]
MAGOH ^1^			
EIF4A3	Richieri-Costa-Pereira syndrome (RCP)	Genetics analysis	[82]
	Anogenital Cancers	Gene expression analysis and RT-PCR	[156]
	Hepatocellular Carcinoma	Gene Expression Profiling Interactive Analysis	[157]
MLN51	HER2+ Breast Cancer	Immunofluorescence analysis	[63]

^1^ MAGOH has not been substantively linked to a human disorder in the academic literature.

## Data Availability

Not applicable.
